# Advanced Characterization of Perovskite Thin Films for Solar Cell Applications Using Time‐Resolved Microwave Photoconductivity and Time‐Resolved Photoluminescence

**DOI:** 10.1002/smtd.202400818

**Published:** 2025-03-18

**Authors:** Emmanuel V. Péan, Jiashang Zhao, Alexander J. Doolin, Rodrigo García‐Rodríguez, Tom J. Savenije, Matthew L. Davies

**Affiliations:** ^1^ SPECIFIC IKC Materials Research Centre, College of Engineering Swansea University Bay Campus Fabian Way Swansea SA1 8EN UK; ^2^ Department of Chemical Engineering Delft University of Technology van der Maasweg 9 Delft 2629 HZ The Netherlands; ^3^ School of Chemistry and Physics University of KwaZulu‐Natal Private Bag X54001 4000 Durban South Africa

**Keywords:** perovskite, solar cells, TRPL, TRMC

## Abstract

Thanks to their direct band‐gap, high absorption coefficient, low manufacturing cost, and relative abundance of component materials, perovskite materials are strong candidates for the next generation of photovoltaic devices. However, their complex photochemistry and photophysics are hindering their development. This is due, in part, to the complex charge carrier recombination pathways in these materials, as well as their instability during measurements. Here, a new characterization methodology is detailed that allows the measurement, with high certainty, of the intrinsic parameters of a single perovskite sample, such as the trap state concentration and carrier mobilities. This methodology is based on a combination of time‐resolved microwave photoconductivity (TRMC) and time‐resolved photoluminescence (TRPL) spectroscopy. Compared to TRPL only, this methodology is faster, does not lead to significant changes in the perovskite properties over time, and increases the certainty of the parameters retrieved. Using this methodology, green solvent systems are studied to replace the traditional harmful solvents usually used when spin–coating perovskites. Although devices made using the greener solvents presented lower efficiencies, TRMC and TRPL measurements highlighted that the perovskites made with these solvents can achieve the same performance compared to the traditional solvent system.

## Graphical abstract

Here a new methodology combining time‐resolved microwave photoconductivity and photoluminescence spectroscopy to measure intrinsic parameters of perovskite samples, namely trap state concentration and carrier mobilities, with high certainty is presented. This faster, more reliable method suggests green solvent systems can achieve comparable performance to traditional solvents despite lower initial efficiencies.

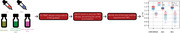

## 1 Introduction

Perovskite solar cells (PSCs) have emerged as a promising technology for the development of low‐cost and highly efficient photovoltaic devices. Over the past decade, PSCs have advanced rapidly, and their energy conversion efficiency has increased from ca. 4% in 2009 to 26.1% in 2024.^[^ ^1^ ^]^ This is comparable to traditional silicon‐based solar cells and higher than other emerging, low‐cost, photovoltaic technologies such as quantum dots solar cells, dye‐sensitized solar cells, and organic solar cells. This progress has largely been achieved through materials and manufacturing developments, as well as a better understanding of the underlying physics of PSCs.^[^ ^2^ ^]^ One of the key advantages of PSCs is their potential low cost of production.^[^ ^3^ ^]^ Unlike traditional silicon‐based solar cells, which require expensive and energy‐intensive manufacturing processes, PSCs can be manufactured using solution‐processing techniques, which are relatively simple and inexpensive.^[^ ^4^ ^]^ Additionally, PSCs can be made using a range of materials, including lead, which is abundant and inexpensive^[^ ^5^, ^6^ ^]^ (although the use of lead can lead to other problems, some of which are addressed in).^[^ ^7^ ^]^ Lead‐based PSCs have demonstrated high power conversion efficiencies, in part, thanks to their high absorption coefficient.^[^ ^8^ ^]^ The band gap of perovskites can be tuned by varying their composition (e.g.,)^[^ ^9^ ^]^ making them useful candidates for tandem solar cells with, e.g., silicon solar cells.^[^ ^10^, ^11^ ^]^

**Figure 1:**
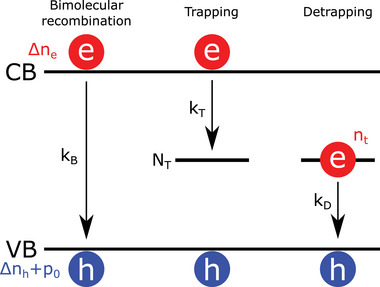
Schematic representation of the Bimolecular‐Trapping–Detrapping model in the case of a *p*‐type material. Photoexcited electrons (concentration Δ*n_e_*) in the conduction band (CB) and holes (concentration Δ*n_h_*) in the valence band (VB) undergo bimolecular recombination at rate constants *k_B_*. Electrons get trapped in trap states (concentration *N_T_*) at a rate constant *k_T_*. Trapped electrons (concentration *n_t_*) can then detrap back to the VB at a rate constant *k_D_*.

Decreasing the open‐circuit voltage (*V*_OC_) loss is crucial to further improve the efficiency of perovskite solar cells in the future. These are usually due to non‐radiative recombination pathways, within the bulk of the perovskite layer or at the charge transport layer/perovskite interfaces. It is therefore essential to understand and quantify the impact of the recombination pathways in perovskite materials. Time‐resolved photoluminescence (TRPL) and time‐resolved microwave photoconductivity (TRMC) allow the study of charge carrier recombination processes in organic and inorganic materials. However, perovskites have been shown to have complex photophysics which makes the investigation of their recombination pathways convoluted.^[^ ^12^, ^13^, ^14^ ^]^ This is partly due to the low exciton binding energy in lead‐based perovskite materials, compared to organic materials used in organic solar cells, leading to a dependence of the recombination process rates on the charge carrier concentration.^[^ ^12^, ^13^ ^]^ Because electrons and holes are uncoupled, their direct recombination requires one entity of each species to happen and is, therefore, a bimolecular process. Similarly, trapping and detrapping can be respectively considered as bimolecular processes between the free electrons and the available trap states, and between the trapped electron and the free hole concentration^[^ ^12^, ^13^ ^]^ (**Figure** **1**).

In the case of a *p*‐type material, the rate equations describing the variations of the photoexcited electron (Δ*n_e_*), photoexcited hole (Δ*n_h_*) and trapped electron (*n_t_*) concentrations are:^[^ ^13^, ^14^ ^]^

(1) $$\frac{d\Delta n_{e}}{dt}=\;G\left(t\right)-k_{B}\Delta n_{e}\left(t\right)\left[\Delta n_{h}\left(t\right)+p_{0}\right]-k_{T}\Delta n_{e}\left(t\right)\left[N_{T}-n_{t}\left(t\right)\right]$$

(2) $$\frac{dn_{t}}{dt}=k_{T}\;\Delta n_{e}\left(t\right)\left[N_{T}-n_{t}\left(t\right)\right]-k_{D}n_{t}\left(t\right)\left[\Delta n_{h}\left(t\right)+p_{0}\right]$$

(3) $$\frac{d\Delta n_{h}}{dt}=\;G\left(t\right)-k_{B}\Delta n_{e}\left(t\right)\left[\Delta n_{h}\left(t\right)+p_{0}\right]-k_{D}n_{t}\left(t\right)\left[\Delta n_{h}\left(t\right)+p_{0}\right]$$

where *k_B_* is the bimolecular recombination rate constant, *k_T_* is the trapping rate constant, *k_D_* is the detrapping rate constant, *p*_0_ is the dark hole concentration, *N_T_* is the trap state concentration and *G* is the carrier generation rate. It is assumed that the carrier concentrations are all zero before photoexcitation, which can be achieved by keeping the sample in the dark. Note that this model can be adapted for *n*‐type materials by simply inverting the notation. In comparison, because of their higher exciton binding energy, recombinations in organic materials tend to be monomolecular, therefore significantly simplifying the above model.^[^ ^12^, ^13^ ^]^

TRPL is a powerful and popular characterization technique to investigate charge carrier recombinations. It consists of measuring the photoluminescence of a sample after a short (typically ps) excitation pulse. TRPL intensity is proportional to the bimolecular recombination rate:

(4) $$I_{TRPL}\left(t\right)\propto k_{B}\Delta n_{e}\left(t\right)\left[\Delta n_{h}\left(t\right)+p_{0}\right]$$

TRPL can be used to characterize organic compounds, allowing to extract carrier lifetime, recombination rate, and carrier extraction rate relatively easily thanks to their excitonic nature. However, as we previously discussed in,^[^ ^12^ ^]^ fitting the TRPL of perovskite materials is a complex task. First, it is essential to allow most charge carriers to recombine between two consecutive excitation pulses to avoid unwanted carrier accumulation during measurements. However, since most detrapping usually happens after the TRPL has reached zero, determining the minimum excitation repetition period preventing carrier accumulation is a complex task. Second, due to the complexity of the charge carrier recombination model (Equations (1), (2), (3), (4)), multiple TRPL decays measured over a wide range of excitation fluences are required to determine with accuracy the different parameter values. Due to their long carrier lifetime and low emission rate, the acquisition of TRPL decays of perovskite can take multiple hours or days. A full set of TRPL decays measured at different excitation fluences can therefore be time‐consuming. Furthermore, long TRPL measurements can lead to changes of the perovskite properties through their interaction with light, oxygen, and moisture amongst others which subsequently affects the TRPL decay shape during the measurements.

TRMC consists of monitoring the sample conductivity changes through the interaction of the photoexcited carriers with microwaves after a short excitation pulse. The variation of the conductivity measured is equal to the sum of the photoexcited electron and hole concentrations multiplied by their respective mobilities (µ_*X*_):

(5) $$I_{TRMC}\;\left(t\right)=\frac{\mu_{e}\Delta n_{e}\left(t\right)+\mu_{h}\Delta n_{h}\left(t\right)}{N_{0}}$$

where *N*_0_ is the photoexcited carrier concentration. Contrary to TRPL, which reaches zero when either carrier concentration is zero, the TRMC intensity only reaches zero when both carrier concentrations are zero. TRMC is thus a more sensitive technique to measure non‐radiative recombination than TRPL. TRMC is also much faster than TRPL and only requires hundreds to thousands of averages to measure a single trace, implying a sample can be fully measured in less than an hour. TRMC usually uses a very low repetition rate excitation source (≈10 Hz) which allows to assume negligible carrier accumulation between excitation pulses.^[^ ^13^ ^]^ Finally, thanks to the short measurement time and low excitation repetition rate, little to no change or degradation is usually observed in the properties of samples during TRMC measurements if the sample is kept in an inert atmosphere.^[^ ^13^ ^]^

Using the parameters retrieved from TRMC or TRPL fitting, it is possible to calculate the quasi‐Fermi level splitting µ_*F*_ as defined by:^[^ ^14^ ^]^

(6) $$\mu_{F}=\frac{kT}{q}\;\ln\left(\frac{\left(\frac{n_{i}^{2}}{p_{0}}+\Delta n_{e}\right)\left(p_{0}+\Delta n_{h}\right)}{n_{i}^{2}}\right)$$

where *k* is the Boltzmann constant, *T* is the temperature, *q* is the elementary charge and *n_i_* is the intrinsic carrier concentration in the sample. The quasi‐Fermi level splitting corresponds to the upper limit of the solar cell open‐circuit voltage and therefore can indicate if the latter can be improved or not.

Although TRMC is more suitable than TRPL to measure perovskite materials and then calculate the quasi‐Fermi level splitting, we show here that the high complexity of charge carrier recombination in perovskite materials requires TRPL measurements to extract meaningful quantities. In,^[^ ^15^ ^]^ the authors created a setup capable of measuring TRPL and TRMC at the same time and under the same measurement conditions, while controlling the measurement temperature, and employ a model that, they claim, can reproduce both the TRPL and TRMC behavior. They used their setup to compare three different perovskite compositions: MAPbI_3_, CsPbBr_3,_ and a mixed‐cation, mixed‐anion perovskite. However, their experimental setup was in the high fluence regime (10^16^ to 10^18^ cm^−3^) and therefore may have overlooked non‐radiative recombinations. In,^[^ ^16^ ^]^ TRMC and TRPL were used to evaluate the effect of 2D layers in a 2D/3D perovskite mixture and employed the mobilities for TRMC and a biexponential fitting for TRPL to conclude that the addition of these 2D perovskites passivates defects on the surface of the perovskite. Here we present a novel methodology that allows one to determine the sample properties, including its quasi‐Fermi level, with high certainty and combines the advantage of quick TRMC measurements and fitting while requiring fewer TRPL measurements than needed when measuring and fitting TRPL alone. Furthermore, it does not require the fitting of TRPL decay curves, which can be computationally costly, when done properly for perovskite materials, due to the necessity to account for carrier accumulation. We then apply this methodology to investigate alternative green solvents to replace the traditional harmful dimethylsulfoxide (DMSO) / dimethylformamide (DMF) solvent system, and ethyl acetate (EA) antisolvent, employing the selection criteria described in previous work;^[^ ^17^ ^]^ namely dimethylpropyleneurea (DMPU), ethanol (EtOH), 2‐methyltetrahydrofuran (2‐MeTHF) and dimethyl carbonate (DMC). While DMPU is still not ideal, it is a significant improvement on the use of DMF and through this approach, the volume of DMPU can be minimized through the use of solvent blends.

## 2 Experimental Section

### 2.1 Sample Fabrication

All fabrication of triple cation perovskite was undertaken with reference to the method reported in.^[^ ^17^ ^]^

#### 2.1.1 Precursor Solution

The Cs_0.066_(MA_0.17_FA_0.83_)_0.934_Pb(I_0.83_Br_0.17_)_3_ precursor solution was manufactured at a molar ratio of 1:1.1:0.2:0.2:0.08 FAI:PbI_2_:PbBr_2_:MABr:CsI using 22.4 mg of methylammonium bromide (Sigma Aldrich, ≥99%), 73.4 mg of lead bromide (Sigma Aldrich, 99.999%), 172 mg of formamidinium iodide (Sigma Aldrich, 99%, anhydrous), and 507.1 mg of lead iodide (TCI Chemicals, 99.99%). For the DMF/DMSO solution, 987 µL of DMF (Acros Organics extra dry, 99%) and DMSO (Acros Organics extra dry, 99%) were added in a 4:1 v/v ratio. The A01 samples include 40 vol% DMSO, 30 vol% DMPU and 30 vol% comprising EtOH and 2‐MeTHF – with 34 vol% 2‐MeTHF to 66 vol% EtOH. The B01 formulation includes 40 vol% DMSO, 30 vol% DMPU and 30 vol% DMC. Subsequently, 53 µL of caesium iodide (Sigma Aldrich, 99.999%) was added using a stock solution (390 mg mL^−1^ in DMSO – Acros Organics extra dry, 99%), which had been previously heated to 100 °C and then cooled to STP. This represents 6.6% Cs addition in a 1.25 m solution. Previous studies have shown high performance for compositions between 5% and 10%.^[^ ^18^ ^]^ This composition includes a PbI_2_ excess.

#### 2.1.2 Films for TRMC and TRPL Measurements

All films were deposited in a freshly purged N_2_ glovebox (GB). GB temperature was regulated to between 22 and 27 °C. Film characterization utilized 2.5 × 1.2 cm^2^ quartz substrates. The quartz substrates were sonicated in acetone and IPA for 10 min respectively, prior to a 10 min UV–ozone treatment. After transfer to an N_2_ GB, 38.4 µL of perovskite precursor was spun at 4000 rpm and 4000 acceleration for 30 s with 96 µL of the anti‐solvent EA or DMC dropped dynamically onto the center of the sample 12 seconds from the start of the spin cycle. A01 and B01 solvent formulations utilized a 2‐step spin coating cycle with 10 seconds at 2000 rpm and 200 rpm m^−1^ acceleration followed by 6000 rpm and 2000 rpm m^−1^ acceleration for 30 seconds with the anti‐solvent added 5 s from the end of the spin cycle (96 µL of anti‐solvent). All films were then annealed on a hot plate at 100 °C for 30 min. All films characterized are coated with poly(methyl methacrylate) (PMMA – Sigma Aldrich, average molecular weight ≈120000 by GPC) under N_2_. PMMA was applied from a 1 m solution in toluene (heated to 80 °C for 12 h), with 100 µL spun at 3000 rpm.

#### 2.1.3 Full Devices

ITO substrates, pre‐cut chemically etched 2.8 cm^2^ pieces of Kintec polished glass (1 mm thick) were first cleaned with 2% Hellmanex solution, before being sonicated for 30 min at STP, followed by 5 min sonication in deionized water. Substrates were transported to a class VI clean room environment for 10 min of sonication in acetone and IPA respectively prior to drying with a nitrogen flow. A 10 min treatment in a plasma cleaner at maximum power using oxygen at 0.3 mbar was conducted before coating. A tin oxide solution was prepared from 15% tin (IV) oxide colloidal solution (Alfa Aesar) which was diluted by a ratio of 3:1 with deionized water and subsequently filtered using 0.2 µm PTFE. This is anticipated to reduce the concentration below 5% but is required to remove agglomerates prone to cause poor surface contact and pinholes. 150 µL of tin oxide solution was spin‐coated onto substrates at 3000 rpm and 3000 acceleration for 30 s. A swab was then used to remove a strip of the SnO_2_ down the middle of the sample, before annealing at 130 °C for 30 min. A ceramic hotplate was used, with the samples left to cool to room temperature before removal. A 10 min UV–ozone treatment was applied to the substrate prior to perovskite deposition. The one‐step deposition method was used to spin–coat perovskite onto the ETL substrates with an anti‐solvent drip of either EA or DMC. For the DMF/DMSO formulation spin coating settings of 4000 rpm, 4000 rpm m^−1^ acceleration for 30 s were used. 250 µL of EA or DMC was dropped onto the sample 12 s from the start of the spin cycle. The A01 and B01 devices were fabricated using a 2‐step spin coating cycle with 10 s at 2000 rpm and 200 rpm m^−1^ acceleration followed by 6000 rpm and 2000 rpm m^−1^ acceleration for 30 s with the anti‐solvent added 5 s from the end of the spin cycle (250 µL of anti‐solvent). Devices were then placed on a hot plate at 100 °C for 30 min to anneal. For the HTL, 85 mg of 2,2′,7,7′‐tetrakis‐(N,N‐di‐4‐methoxyphenylamino)‐9,9′‐spirobifluorene (Spiro‐OMeTAD – Merck Millipore – Sigma Aldrich) was dissolved in 996 µL of chlorobenzene (anhydrous, 99.8%) 34 µL 4‐tertbutylpyrridine, 20 µL of bis(trifluoromethane)sulfonimide lithium salt (Li‐TFSI) in acetonitrile (520 mg Li‐TFSI in 1 mL acetonitrile (anhydrous, 99.8%)), and 8 µL of tris(2‐(1H‐pyrazol‐1‐yl)−4‐tert‐butylpyridine)‐cobalt(III)tris(bis(trifluoromethylsulfonyl)imide)) (FK‐209) were subsequently added. The completed hole transport layer (HTL) solution was then filtered with 0.2 µm PTFE filter before deposition. 100 µL of Spiro‐OMeTAD solution was spread onto the PAL, with a 30 s spin cycle at 4000 rpm. Oxidation of the HTL was achieved by leaving the device in a dark environment for 12–24 h. Finally, gold wire (Au, 99.99% purity 1.0 mm thick Sigma Aldrich) was used to deposit top contacts on all devices using an Edwards bell jar evaporator at a pressure of 10^−5^ mbar.

### 2.2 Sample Characterization

TRPL was measured using an Edinburgh Instruments Lifespec time‐correlated single photon counting and an EPL405 laser (405 nm excitation wavelength, 100 kHz repetition rate, 80 ps pulse width).

TRMC was measured using an in‐house setup. Excitation was provided by an Ekspla Nd:YAG pulsed laser with a 650 nm excitation wavelength, a 3.5 ns pulse width with a 10 Hz repetition rate. High excitation fluence measurements were carried out using an open‐cell microwave cell, while low fluence measurements used a resonant cavity microwave cell with a response time of 18 ns. Both cells are air‐tight and allow to keep the samples in nitrogen atmosphere during the measurements, to prevent reaction with oxygen and moisture. The combination of both cells allows laser intensity variation over more than 3 orders of magnitude. A 8.5 GHz –100 mW voltage‐controlled oscillator was used to generate the microwaves.^[^ ^13^ ^]^

Film thickness was measured using a Dektak profilometer (model Veeco).

Sample absorptance was measured using a Perkin‐Elmer UV/Vis spectrophotometer (Lambda 1050) fitted with an integrating sphere.

### 2.3 TRMC and TRPL Simulations

The Bimolecular‐Trapping–Detrapping model discussed above was used to fit the TRMC data and simulate TRPL. For TRMC, a Gaussian pulse is used for the generation rate *G*:

(7) $$G\;\left(t\right)=\;Ae^{-\frac{4\log\left(2\right){\left(t-t_{0}\right)}^{2}}{w^{2}}}$$

where *A* is the amplitude, *t*_0_ is the centre of the Gaussian and *w* is the pulse width. For resonant cavity measurements, the simulated TRMC intensity was reconvolved with the cavity response function defined by:

(8) $$IRF\;\left(t\right)=e^{-\frac{t}{\tau }}\;$$

where τ is the response time of the cavity.

For TRPL simulations, thanks to the much shorter excitation pulse (≈80 ps), it is simply assumed that the charge carriers are instantaneously generated at *t*  =  0. The photoexcited concentration after a certain excitation pulse *p* is thus equal to the sum of the photoexcited carriers by the pulse *N*_0_, and any carrier present in the system just before the pulse:^[^ ^12^ ^]^

(9) $$\Delta n_{e}^{p}\;\left(t\;=\;0\right)=N_{0}\;+\Delta n_{e}^{p-1}\left(t\;=\;T\right)$$

(10) $$\Delta n_{h}^{p}\;\left(t\;=\;0\right)=N_{0}\;+\Delta n_{h}^{p-1}\left(t\;=\;T\right)$$

(11) $$\Delta n_{t}^{p}\;\left(t=0\right)=\Delta n_{t}^{p-1}\left(t=T\right)$$

(12) $$G\;\left(t\right)=\;0$$

where *T* is the excitation repetition period. The photoexcited concentration *N*_0_ was calculated from the laser fluence *I*_0_ the sample absorptance *A* at the excitation wavelength, and the sample thickness *d*:^[^ ^12^ ^]^

(13) $$N_{0}=\frac{I_{0}A}{d}\;$$

Using the above equations, it is possible to simulate the TRPL after multiple excitation pulses, thus allowing carrier accumulation to be accounted for in the sample. As discussed in a previous paper, experimental TRPL decays resulting from billions of excitation pulses can be simulated with only a few hundred by defining a stabilized pulse after which the carrier concentration variations are similar:^[^ ^12^ ^]^

(14) $$\left|n_{X}^{p-1}\left(t\right)-n_{X}^{p}\left(t\right)\right|\leq 10^{-6}N_{0}$$

Since a 10 Hz excitation repetition rate was used for TRMC measurements, it is assumed that carrier accumulation is negligible during these measurements and therefore the TRMC intensity was calculated with a single excitation pulse. In both cases, the carrier concentrations are assumed zero before excitation.

The quasi‐Fermi level µ_*F*_ was calculated at room temperature and the intrinsic carrier concentration was taken from^[^ ^19^ ^]^ and measured from a similar perovskite composition (*n_i_* =  1.26 × 10^5^ *cm* ^−3^).

### 2.4 Data Fitting

TRMC fitting was carried out using a non‐linear least square optimization. For a dataset containing *M* curves, each curve *i* containing *N_i_* datapoints, the optimization residue *SS_res_* is:

(15) $$S\;S_{res}=\sum_{i}^{M}\sum_{j}^{N_{i}}{\left(y_{i,j}-F\left(t_{i,j},A_{i}\right)\right)}^{2}$$

where *y*_*i*,*j*_ is the experimental TRMC intensity at the time *t*_*i*,*j*_ of point *j* of curve *i*. *A_i_* are the model parameters associated with curve *i*, and *F* is the fitting model given by:

(16) $$F\;\left(t,\text{…}\right)=I_{TRMC}\;\left(t,G^{i},k_{B}^{i},\text{…}\right)$$

where *I_TRMC_* are calculated using the above model. The quality of the fit is estimated from the coefficient of determination *R* ^2^:

(17) $$R^{2}=\;1-\frac{SS_{res}}{SS_{total}}$$

where *SS_total_* is defined as the sum of the squared difference between each point and the average of all curves $\bar{y}$:

(18) $$S\;S_{total}=\sum_{i}^{M}\sum_{j}^{N_{i}}{\left(y_{i,j}-\bar{y}\right)}^{2}\;$$

The least‐square optimization was solved using a trust region reflective algorithm as implemented by the SciPy package.^[^ ^20^ ^]^ Each set of TRMC decays measured at different excitation fluences was fitted using a global approach by using the same set of parameter values, only varying the generation rate *G*.

**Figure 2:**
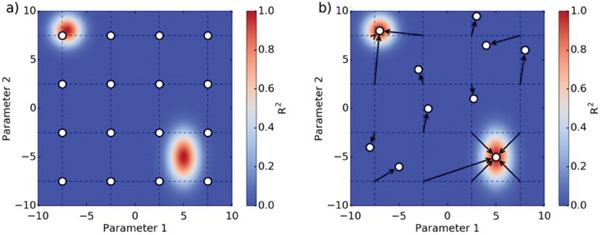
Example of the grid fitting analysis to find local minima based on a model with two parameters. The associated *R* ^2^ value is displayed in the background to show which parameter values yield a good fit. a) A grid of guess values is created as shown by the white dots. b) The fitting optimization is carried out with each set of guess values. Here, 3 and 5 fits converge toward two distinct solutions while the remaining optimizations yield incorrect solutions as indicated by their low *R* ^2^. Due to the high computational cost of this method, we did not perform a formal convergence test for the grid points. However, the fact that most optimizations converged toward 2–3 distinct solutions suggests that our grid was likely sufficient.

In order to find all the local minima of the fitting optimization, a grid of guess values are generated using *k_B_* = 10^−11^ ,10^−10^ *cm* ^3^/*s*, *k_T_* = 10^−9^ ,10^−8^ *cm* ^3^/*s*, *k_D_* = 10^−11^ ,10^−10^ *cm* ^3^/*s*, *N_T_* = 10^12^ , 10^13^ *cm* ^−3^, *p*_0_ = 10^12^ , 10^13^ *cm* ^−3^, µ_*e*_ =  1,  10 *cm* ^2^/(*Vs*) and µ_*h*_ =  1,  10 *cm* ^2^/(*Vs*). Fitting is then carried out using each set of guess values. Only good fits are then considered (i.e., *R* ^2^ > 0.9), and fits converging toward the same minimum are merged into one. An example of the “grid fitting” analysis is shown in **Figure** **2** for a model with two parameters.

## 3 Results & Discussion

**Figure 3:**
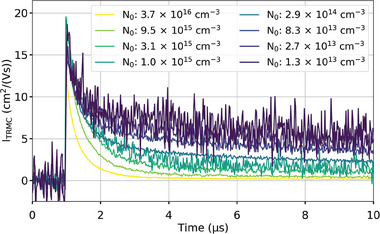
TRMC decays of a triple cation perovskite thin film measured at different excitation fluences.

**Figure** **3** shows the TRMC decays of a triple‐cation thin film measured at different excitation fluences, generating between 1.3 × 10^13^ *cm* ^−3^ and 3.6 × 10^16^ *cm* ^−3^ charge carrier concentrations (see Table S1 for the sample thickness, Figure S1 for its absorptance, and Figure S2 for its XRD pattern, Supporting Information). As predicted by the model, as the laser fluence is decreased, the decay lifetime increases due to the lower bimolecular recombination contribution and the higher contribution of trapping and detrapping.

**Figure 4:**
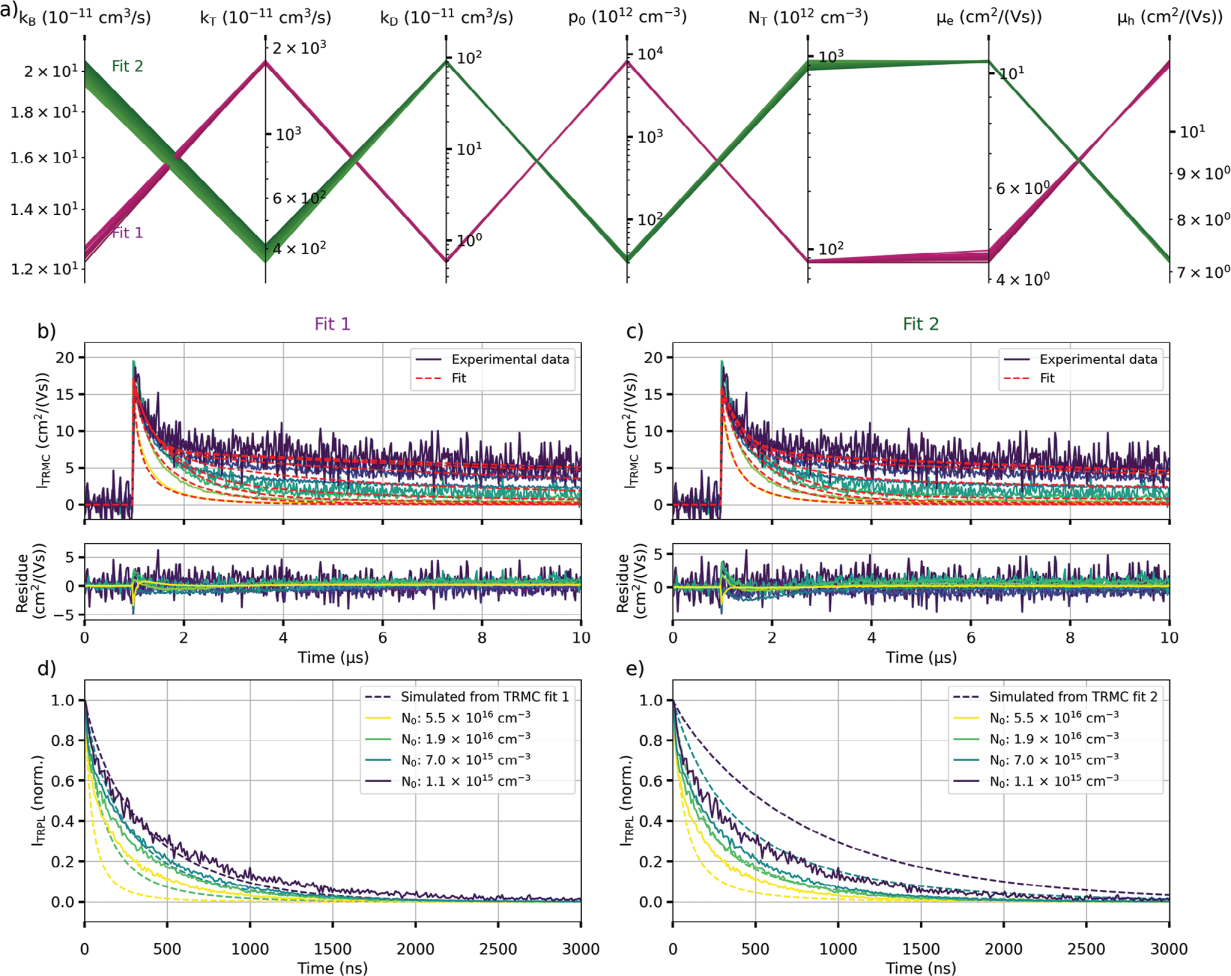
Results of the grid fitting analysis. a) Parallel coordinate plot showing the different optimized parameter values obtained. b) & c) Comparison of the two fitting solutions obtained and their residues. d) & e) Comparison between the experimental TRPL and the TRPL simulated using the two sets of variables fitting the TRMC traces well.

In order to find all the sets of recombination parameters able to fit these data, we fitted these data using the Bimolecular‐Trapping–Detrapping model previously discussed with a wide range of guess parameter values. For each parameter, two guess values were considered, yielding 128 combinations. Each combination of guess values was then used to fit the data. Out of 128 fitting optimizations, 116 were considered good fits which parameters are displayed in a parallel coordinate graph (**Figure** **4**a).

The fitting optimizations converged toward two distinct solutions (Figure 4b,c). Both solutions fit the data well as shown by their small residue. We note that the residue spikes at high excitation fluences likely originate from fluctuations of the laser power during the measurements. Both solutions have very similar bimolecular recombination rate constant *k_B_* but significantly differ for other parameters (Figure 4a). To determine the correct solution, the TRPL intensity was simulated from these two solutions and was compared to experimentally measured TRPL (Figure 4d,e). For each solution, the model parameters were kept the same, except for the excitation fluence which was changed to account for the much shorter TRPL pulse (Equation 12). Furthermore, we also accounted for charge carrier accumulation due to the higher excitation repetition rate of TRPL, by simulating the carrier concentration after multiple excitation pulses. As shown in Figure 4d, the TRPL simulated from the first solution matches well the experimental TRPL at low fluence but starts to differ at higher fluence. On the contrary, the simulated TRPL from the second solution does not match the experimental TRPL except at one fluence (Figure 4e). It is worth mentioning that based on the Saha equation, not all photons yield charge carriers at high excitation fluences.^[^ ^21^ ^]^ We know that this phenomenon is not happening for the fluences used during our TRMC measurements as the measured carrier mobility sum did not change depending on the fluence. However, we are potentially overestimating the charge carrier concentration at the highest excitation fluence used to measure TRPL, which is higher than the highest fluence used during TRMC measurements. This would therefore explain why the simulated TRPL from Fit 1 decays faster compared to the experimental TRPL.

We can therefore say with high certainty that Fit 1 represents our triple‐cation perovskite sample whose parameters are shown in **Table** **1**. A similar trap state concentration was previously calculated in^[^ ^14^, ^22^ ^]^ for a similar perovskite composition ((Cs_0.06_FA_0.79_MA_0.15_)Pb(I_0.85_Br_0.15_)_3_). However, the values previously reported showed a higher carrier mobility sum (µ_*e*_ + µ_*h*_ ≈ 40 *cm* ^2^/(*Vs*)) and bimolecular recombination rate constant (*k_B_* =  40 × 10^−10^ *cm* ^3^/*s*). It is also worth mentioning that the charge carrier recombination model used here does not differentiate between *n*‐type and *p*‐type doping. It is therefore possible that the doping concentration would correspond to *p*‐type doping instead of the *n*‐type doping previously mentioned (in which case, we would consider hole trapping).

**Table 1:** Parameter values extracted from the TRMC and TRPL measurements.

Parameter	Value
*k_B_*	2.1 × 10^−10^ *cm* ^3^/*s*
*k_T_*	4.2 × 10^−9^ *cm* ^3^/*s*
*k_D_*	9.2 × 10^−10^ *cm* ^3^/*s*
*N_T_*	8.4 × 10^14^ *cm* ^−3^
*p*_0_	3.2 × 10^13^ *cm* ^−3^
µ_*e*_	10.3 *cm* ^2^/(*Vs*)
µ_*h*_	7.2 *cm* ^2^/(*Vs*)

**Figure 5:**
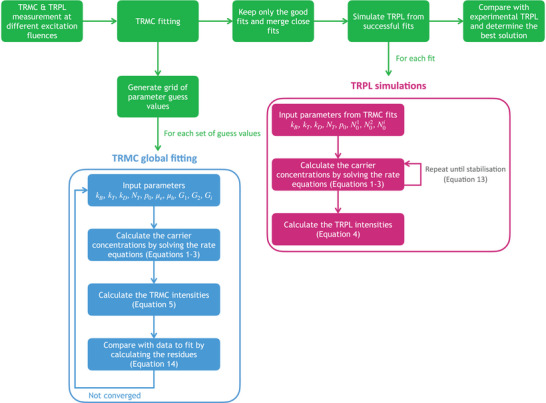
Schematic representation of our new methodology.

Our methodology is schematically summarized in **Figure** **5**.

**Figure 6:**
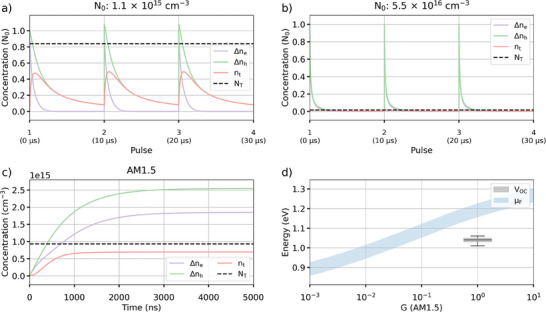
Analysis of the fitting result. Variations of the carrier concentrations after multiple excitation pulses under a) medium ($N_{0}∼N_{T}$) and b) high (*N*_0_ > *N_T_*) excitation fluence during TRPL measurements. c) Variation of the carrier concentrations under AM1.5 Direct illumination and d) calculated quasi‐Fermi level (µ_*F*_) for increasing generation rate compared to the experimental open‐circuit voltage (*V_OC_*). The area shows the error on µ_*F*_ dependent upon the intrinsic carrier concentration between 6.25 × 10^4^ *cm* ^−3^ and 25 × 10^4^ *cm* ^−3^.

Analysis of fitting solution one shows that, although a significant number of trapped electrons and holes are left in the trap‐states and Valence band before the next excitation pulse, carrier accumulation here is very limited and leads to only 2% difference between the TRPL decays obtained after a single excitation pulse and multiple pulses (**Figure** **6**a).

At the highest fluence used during the TRPL measurements, the trap states are fully saturated, and carrier accumulation is negligible (Figure 6b). Using a constant generation rate matching AM1.5 Direct illumination (*G*  =  2.5 × 10^12^ *cm* ^−3^/*ns*) shows that the trap states get quickly saturated and most recombination are direct (Figure 6c). Finally, we simulate the quasi‐Fermi level using Equation 6 and compare it to the value obtained from devices made using our perovskite system (Figure 6d). We note that we obtained a *V*_OC_ of 1.05 V, similar to the one reported by Abdi‐Jalebi in^[^ ^22^ ^]^ for a similar perovskite composition (CsMAFAPb(I_0.85_Br_0.15_)_3_). To account for potential variations of the intrinsic carrier concentration in our sample compared to the one reported in,^[^ ^19^ ^]^ we estimated the quasi‐Fermi level between 0.5*n_i_* and 2*n_i_*. Compared to the experimental *V*_OC_, we see a difference from 0.12 up to 0.2 eV with the quasi‐Fermi level. This shows that the efficiency of these devices could be significantly improved by, for example, passivating trap states at the transport layer interfaces, such as using K^+^ passivation which showed a significant improvement of the *V*_OC_ in.^[^ ^14^ ^]^

We used this method to investigate different solvent systems (DMF/DMSO, DMSO/DMPU/EtOH/2‐MeTHF and DMSO/DMPU/DMC herein referred as A01 and B01) and antisolvents (ethyl acetate and dimethyl carbonate – herein referred to as EA and DMC) (**Figure** **7**; Figures S3–S15, Supporting Information).

**Figure 7:**
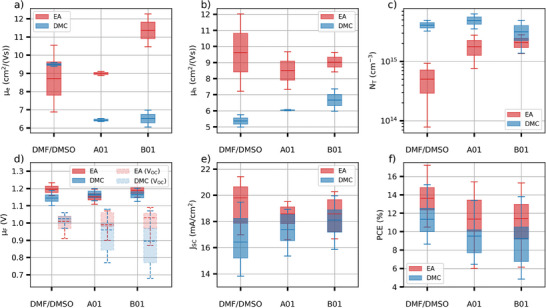
Properties of perovskites made using the DMF/DMSO, A01, and B01 solvent solvents, and the Ethyl acetate (EA) and dimethyl carbonate DMC anti‐solvents. a) Electron and b) hole mobilities. c) Trap state concentration d) Quasi‐Fermi level and open‐circuit voltage (*V_OC_*) of devices (dashed lines) using the same solvent systems. e) Short‐circuit current (*J_SC_*) and f) power conversion efficiency (PCE).

Perovskite films made using the DMC anti‐solvent present equal or lower electron and hole mobilities compared to EA (Figure 7a,b). This is likely responsible for the lower short‐circuit current measured in devices made with this anti‐solvent (Figure 7e; Figure S15, Supporting Information). Although a slight increase of the hole mobility is measured when using A01 and B01, the electron mobility significantly decreases compared to DMF/DMSO. The mobility sum is therefore lower for A01 and B01 while the associated short‐circuit current is higher than DMF/DMSO. We hypothesize that the low hole mobility limits the short‐circuit current in the DMF/DMSO‐DMC perovskite. The lower discrepancy between the electron and hole concentrations as well as the higher hole mobility could therefore explain the higher short‐circuit current observed in these perovskites. DMC perovskites also present a higher trap state concentration compared to EA, consistent with the lower open‐circuit voltage (Figure 7d). Little to no difference in terms of trap state concentration is observed between the different solvent systems when using DMC. Overall, a lower power conversion efficiency is measured for devices made using the DMC anti‐solvent compared to EA (Figure 7f). A01 and B01 perovskites present similar efficiencies while being 2.3 points lower than DMF/DMSO.

Using the EA anti‐solvent, while the hole mobility appears unaffected by the solvent system, a higher electron mobility is measured when using B01. However, this does not result in a higher short‐circuit current, which is consistent with the short‐circuit current being limited by the hole mobility. The trap concentration is 4 times higher in A01 and B01 compared to DMF/DMSO, however, this does not seem to affect the open‐circuit voltage significantly. The lower efficiency measured with A01 and B01 compared to DMF/DMSO can be explained by the lower fill factor in these devices (Figure S16, Supporting Information).

Although the triple cation perovskite fabricated with the greener solvent systems tends to present slightly lower performances than the more widely used DMF/DMSO solvent system with EA anti‐solvent, our simulations show that the quasi‐Fermi level does not significantly depend on the solvent system, or the anti‐solvent used during manufacturing (Figure 7d). In particular, while the A01 and B01 solvent systems, and the DMC anti‐solvent present a higher trap state concentration, it is compensated by a lower trapping rate constant for these (Figure S17, Supporting Information). This suggests that these greener alternative solvents are therefore adequate replacement for DMF/DMSO‐EA and might simply need more refinement, through tuning the solvent blends, to achieve similar performances. It is interesting to note that these solvent systems have matched the performance of DMF/DMSO solvent systems with a MAPbI_3_ absorber layer and thus this implies there is a requirement to alter solvent blends to a particular perovskite precursor to optimize the performance in the triple cation perovskite.^[^ ^17^ ^]^

## 4 Conclusion

We have presented a novel characterization methodology to confidently measure the intrinsic properties of perovskite thin films. Our process starts with the measurement of the TRMC and TRPL of the sample studied at different excitation fluences. The TRMC decays are then fitted with a state‐of‐the‐art charge carrier recombination model and a global fitting approach. Different sets of guess values for the different parameters of the models are used, allowing to find the different local minima of the optimization. The fitting solutions are then curated and grouped up. Out of the few solutions left, we simulate the TRPL decays based on the experimental measurement parameters. We then compare the simulated TRPL decays to the experimentally measured decays, accounting for the different excitation conditions between TRPL and TRMC, allowing us to determine the best solution representing the sample properties. This method offers many advantages compared to traditional characterization methods such as TRMC or TRPL measurements only. First, while measuring the TRPL only would require exposing the sample to light for a long time to acquire all the decays required, our method limits the exposure of the sample because of the low excitation repetition rate of TRMC and the fact that we do not require to measure the TRPL with as many fluences. Second, while carrier accumulation can be assumed negligible during TRMC measurements, it is almost unavoidable during TRPL measurements. Fitting TRPL considering carrier accumulation is computationally costly due to the requirement to simulate hundreds of excitation pulses instead of just one. Here, we fit TRMC data without considering carrier accumulation and only consider it when simulating the TRPL, thus greatly reducing the computational cost compared to fitting TRPL. Our method is therefore faster, less likely to induce changes to the sample during the measurements, less computationally intensive, and increases the confidence in the parameter values retrieved compared to TRMC measurements only. We have investigated potential green solvent replacements for dimethyl formamide, dimethyl sulfoxide, and ethyl acetate. Our findings showed that, although devices made using our greener alternatives presented lower open‐circuit voltages – and therefore lower efficiencies, they could achieve the same potential maximum open‐circuit voltage.

## Conflict of Interest

The authors declare no conflict of interest.

## Supporting information



Supporting Information

## Data Availability

The data that support the findings of this study are available from the corresponding author upon reasonable request.
