# Supplementary material for: Advanced Characterization of Perovskite Thin Films for Solar Cell Applications Using Time‐Resolved Microwave Photoconductivity and Time‐Resolved Photoluminescence
Source: Small Methods. 2025 Mar 18;9(4):2400818. doi: 10.1002/smtd.202400818 (PMC12020338; doi:10.1002/smtd.202400818)
Supplement: Supplementary file 1 — Supporting Information [file SMTD-9-2400818-s001.docx]

# Advanced characterisation of perovskite thin films using time-resolved microwave photoconductivity and time-resolved photoluminescence

Emmanuel V. Péan^1^, Jiashang Zhao^2^, Alexander J. Doolin^1^, Tom J. Savenije^2^, Matthew L. Davies^1,3^

^1^SPECIFIC IKC, Materials Research Centre, College of Engineering, Swansea University Bay Campus, Fabian Way, Swansea SA1 8EN, UK
^2^Department of Chemical Engineering, Delft University of Technology, van der Maasweg 9, 2629 HZ Delft, The Netherlands
^3^School of Chemistry and Physics, University of KwaZulu-Natal, Durban, RSA

**Figure S1**: Sample absorptance. The line corresponds to the average between the different samples, the area is the spread.

**Table S1**: Sample thicknesses measured via profilometry.

| **Solvent** | **Anti solvent** | **Thickness (nm)** |
| --- | --- | --- |
| DMF/DMSO | EA | 530 |
|  | DMC | 482 |
| A01 | EA | 565 |
|  | DMC | 532 |
| B01 | EA | 533 |
|  | DMC | 529 |


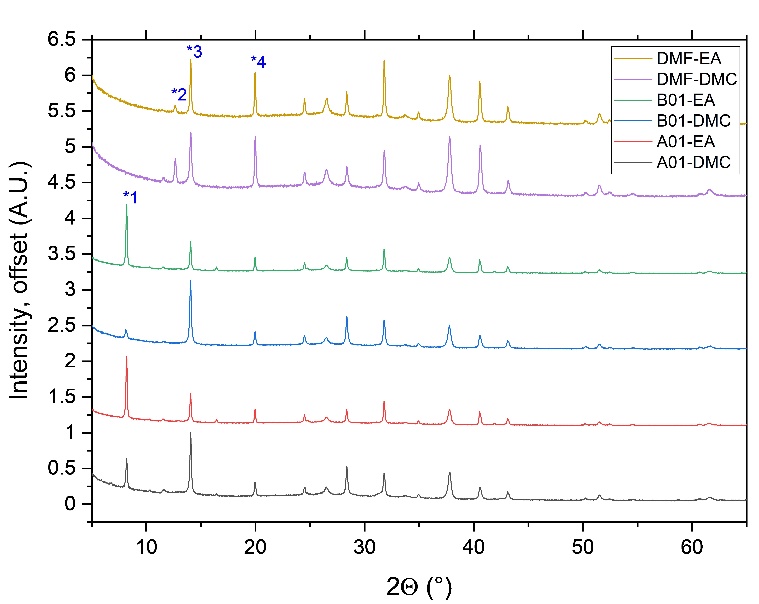


**Figure S2**: XRD pattern of samples with the same composition presented in this work, where DMF, B01 and A01 represent a different solvent system, and EA and DMC represent the antisolvent employed. 4 main peaks are highlighted: 8.2° probably due to the presence of a 2D layer, [10.1039/d1gc00079a, 10.1039/C8TA05475D] 12.6° corresponding to the presence of PbI_2_ [ 10.1039/d1gc00079a] and the 14.1° and 20.0°, corresponding to the perovskite crystal orientations (110) and (100), respectively. [10.1016/j.isci.2024.109306, 10.1039/d1gc00079a].

**Figure S3**: Fitting of the TRMC of the triple cation perovskite using the solvent system B01 and DMC antisolvent (sample 1). **a)** All fitting parameters obtained, **b)** R^2^ value for the different fits and **c­)** & **d)** fit (dashed lines) overlayed on top of the experimental data (full lines).

**Figure S4**: Fitting of the TRMC of the triple cation perovskite using the solvent system B01 and DMC antisolvent (sample 2). **a)** All fitting parameters obtained, **b)** R^2^ value for the different fits and **c­)** & **d)** fit (dashed lines) overlayed on top of the experimental data (full lines).

**Figure S5**: Fitting of the TRMC of the triple cation perovskite using the solvent system A01 and DMC antisolvent (sample 1). **a)** All fitting parameters obtained, **b)** R^2^ value for the different fits and **c­)** & **d)** fit (dashed lines) overlayed on top of the experimental data (full lines).

**Figure S6**: Fitting of the TRMC of the triple cation perovskite using the solvent system A01 and DMC antisolvent (sample 2). **a)** All fitting parameters obtained, **b)** R^2^ value for the different fits and **c­)** & **d)** fit (dashed lines) overlayed on top of the experimental data (full lines).

**Figure S7**: Fitting of the TRMC of the triple cation perovskite using the solvent system A01 and EA antisolvent (sample 1). **a)** All fitting parameters obtained, **b)** R^2^ value for the different fits and **c­)** & **d)** fit (dashed lines) overlayed on top of the experimental data (full lines).

**Figure S8**: Fitting of the TRMC of the triple cation perovskite using the solvent system A01 and EA antisolvent (sample 2). **a)** All fitting parameters obtained, **b)** R^2^ value for the different fits and **c­)** & **d)** fit (dashed lines) overlayed on top of the experimental data (full lines).

**Figure S9**: Fitting of the TRMC of the triple cation perovskite using the solvent system DMF/DMSO and EA antisolvent (sample 1). **a)** All fitting parameters obtained, **b)** R^2^ value for the different fits and **c­)** & **d)** fit (dashed lines) overlayed on top of the experimental data (full lines).

**Figure S10**: Fitting of the TRMC of the triple cation perovskite using the solvent system DMF/DMSO and EA antisolvent (sample 2). **a)** All fitting parameters obtained, **b)** R^2^ value for the different fits and **c­)** & **d)** fit (dashed lines) overlayed on top of the experimental data (full lines).

**Figure S11**: Fitting of the TRMC of the triple cation perovskite using the solvent system B01 and EA antisolvent (sample 1). **a)** All fitting parameters obtained, **b)** R^2^ value for the different fits and **c­)** & **d)** fit (dashed lines) overlayed on top of the experimental data (full lines).

**Figure S12**: Fitting of the TRMC of the triple cation perovskite using the solvent system B01 and EA antisolvent (sample 2). **a)** All fitting parameters obtained, **b)** R^2^ value for the different fits and **c­)** & **d)** fit (dashed lines) overlayed on top of the experimental data (full lines).

**Figure S13**: Fitting of the TRMC of the triple cation perovskite using the solvent system DMF/DMSO and DMC antisolvent (sample 1). **a)** All fitting parameters obtained, **b)** R^2^ value for the different fits and **c­)** & **d)** fit (dashed lines) overlayed on top of the experimental data (full lines).

**Figure S14**: Fitting of the TRMC of the triple cation perovskite using the solvent system DMF/DMSO and DMC antisolvent (sample 2). **a)** All fitting parameters obtained, **b)** R^2^ value for the different fits and **c­)** & **d)** fit (dashed lines) overlayed on top of the experimental data (full lines).

**Figure S15**: Forward and reverse IV of devices made using the different solvent systems. The area under the curve represents the error.

**Figure S16**: Fill factor of devices made using the different solvent systems.

**Figure S17**: Trapping ($k_{T}$) and detrapping ($k_{D}$) rate constants measured for the different solvent systems.
